# Spontaneous Intramuscular Hemorrhage in Anti-MDA5 Positive Dermatomyositis: A Case Series and Literature Review

**DOI:** 10.3389/fmed.2021.802753

**Published:** 2022-01-24

**Authors:** Zhangling Xu, Xia Lv, Wenwen Xu, Yan Ye, Xiaodong Wang, Shuang Ye, Huihua Ding, Wanlong Wu

**Affiliations:** Department of Rheumatology, Renji Hospital, Shanghai Jiaotong University School of Medicine, Shanghai, China

**Keywords:** dermatomyositis, spontaneous intramuscular hemorrhage, anti-MDA5 antibody, case series, prognosis

## Abstract

**Objective:**

Spontaneous intramuscular hemorrhage (SIH) is a rare but life-threatening complication associated with dermatomyositis (DM). This study reported a case series of SIH associated with DM. In addition, the characteristics and prognostic effects for this complication were analyzed based on literature review.

**Methods:**

We reported seven cases of anti-melanoma differentiation-associated gene five positive dermatomyositis (MDA5^+^DM) complicated by SIH in our single-center cohort, and a comprehensive literature review was performed. Clinical characteristics, treatment, and outcome data of all eligible reported cases were summarized. Potential prognostic effects were identified by comparisons between the deceased and survivors.

**Results:**

Among cumulatively reported patients with DM patients and SIH, the overall mortality was 60.9% (14/23) (including our cases). Fourteen out of nineteen (73.7%) hemorrhagic events occurred within 6 months of disease onset. Anti-MDA5 antibody predominated in those myositis-specific antibodies available cases (8/10), although patients with positive anti-NXP2 and anti-Mi2 have also been documented. Iliopsoas (52.2%, 12/23) was the most frequently involved bleeding location. Bleeding in deep muscles was identified to be associated with poorer prognosis. The mortality of patients with DM and deep muscular hematoma (non-palpable) (80%, 12/15) was significantly higher than that of patients with only superficial muscular hematoma (palpable) (25%, 2/8) (*p* =0.023).

**Conclusion:**

Spontaneous hematoma in non-palpable deep muscles probably leads to excess mortality in dermatomyositis, particularly for those with anti-MDA5 antibody, which often occurs within 6 months of disease onset. Clinicians should be vigilant to this rare but potentially fatal complication and carefully balance the risks and benefits of prophylactic anti-thrombotic treatment.

## Introduction

Dermatomyositis (DM) is a heterogeneous group of systemic autoimmune rheumatic disorders with characteristic cutaneous manifestations and symmetrical proximal myositis. Visceral involvement including interstitial lung disease (ILD) and constitutional symptoms such as fever are also common in DM. Myositis-specific antibodies (MSAs) help to define distinct clinical phenotypes of organ involvement and may provide assistance in diagnosis, treatment, and prognostication. Anti-melanoma differentiation-associated gene five positive dermatomyositis (MDA5^+^DM) is a subtype of DM characterized by typical rashes, without or mild myositis but notable ILD, frequently as a rapidly progressive course, with prominently high 6-month mortality reported varying from 33 to 66% because of respiratory failure (1–6).

In contrast, spontaneous intramuscular hemorrhage (SIH) is a rare, neglected but life-threatening complication of DM. Until now, little is known about DM-associated SIH. The pathogenetic mechanism of intramuscular hematoma remains unclear, and previous case reports have speculated on a possible relationship with active inflammation of muscle-supplying vessels, empirical prophylaxis anti-thrombotic therapy, and high-dose glucocorticoid use (7). In addition, no previous study has reported on the incidence or investigated the prognostic effects of this rare complication. Here, we report seven patients diagnosed with MDA5^+^DM complicated by SIH in our single-center cohort. A comprehensive literature review was then performed, and the prognostic effects of this complication were analyzed.

## Cases Presentation

### Case 1 (Listed as Case No. 17)

A 41-year-old male patient presented with rashes, swollen and painful joints, high fever, myalgia, generalized fatigue, and exertional dyspnea for 1 month. Physical examination showed Gottron's papules on bilateral metacarpophalangeal joints and swelling of interphalangeal joints. Muscle weakness was not detected by manual muscle testing. MSAs and MAAs tests revealed double positivity of anti-MDA5 antibody and anti-Ro52 antibody by Euroline immunoblotting assay, while antinuclear antibody (ANA), extractable nuclear antigen (ENA), and anti-neutrophil cytoplasmic antibodies (ANCAs) were all negative. Positive laboratory findings included elevated creatinine kinase (CK, 978 U/L; upper limit of normal (ULN): 200 U/L), alanine aminotransferase (ALT, 508 U/L; ULN: 40 U/L), ferritin (6,849 ng/ml; ULN: 336 ng/ml), and troponin I (0.14 ng/ml, ULN:0.04 ng/ml). Hemoglobin (HB) (127 g/L) was slightly decreased. Platelet count and coagulation function were both normal. Electrocardiogram (ECG) test was normal. Pulmonary high-resolution computed tomography (HRCT) revealed ground-glass opacities and consolidation in bilateral lower lung lobes. The partial pressure of oxygen in arterial blood gas (PaO_2_) at room air was 81 mmHg. Pulmonary function tests showed a mild restrictive ventilatory defect (forced vital capacity, FVC, predicted: 75.6%). Skin biopsy revealed loose collagen and lymphocyte infiltration around capillaries in the superficial and middle dermis. Positron emission tomography-computed tomography (PET-CT) showed ILD changes and inflammation of retroperitoneal muscles without evidence of malignancy.

He was, thus, diagnosed with MDA5^+^DM complicated by ILD. Immunosuppressive treatment including intravenous methylprednisolone (80 mg per day), oral tofacitinib (5 mg twice daily), and intravenous immunoglobulin (10 g per day for 3 days) were prescribed due to active disease. Although he had no chest pain, abnormal ECG, or previous history of coronary heart disease, treatment with prophylactic antiplatelet therapy (aspirin 100 mg per day) was initiated owing to the elevation of troponin I to.92 ng/ml 1 week later.

**Figure 1:**
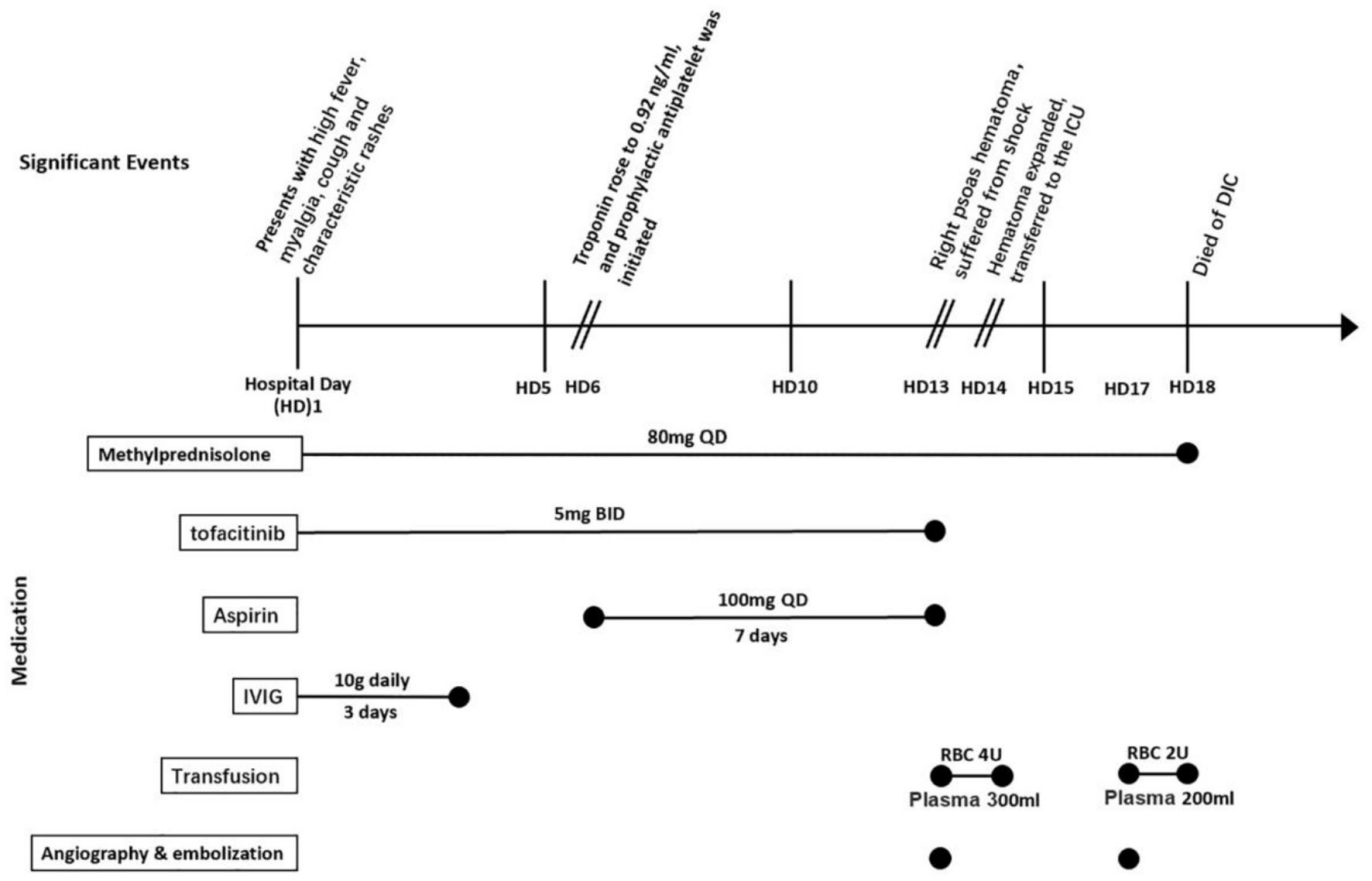
Timeline of significant events and medication regimen for presented case 1 (listed as case no. 17). ICU, intensive care unit; DIC, disseminated intravascular coagulation; IVIG, intravenous immunoglobulin.

However, 13 days after admission, he suddenly complained pain in the right lower back, without trauma. Complete blood cell (CBC) test showed a decline in HB to 65 g/L. CT scan revealed a new-onset hematoma in the right psoas major extending down to the right hip joint space. Subsequent emergent angiography confirmed active bleeding from the right lumbar artery. Coil embolization was immediately implemented, together with active fluid resuscitation and blood transfusion. Then, he was transferred to the intensive care unit (ICU). However, he again suffered hemorrhagic shock only several hours later, and HB dropped to 42 g/L. Repeated CT confirmed the expansion of the existing hematoma. A second angiography revealed new bleeding from collateral circulations of right internal iliac artery, and coil embolization was carried out again. Unfortunately, the patient eventually died of disseminated intravascular coagulation (DIC) on day 18 (Figure 1).

### Case 2 (Listed as Case No. 18)

A 66-year-old female patient presented to our center with 2-month exertional dyspnea, 1-month bilateral periorbital rashes, and myalgia. Notable features of the physical examination were heliotrope rash, mechanic's hand, edema on the face, and velcro rales in chest auscultation. Laboratory tests showed elevation of erythrocyte sedimentation rate (ESR, 51 mm/H, ULN: 20 mm/H), CK (344 U/L, ULN: 200 U/L), and ferritin (1,660 ng/ml, ULN: 336 ng/ml). HB level was 104 g/L. Coagulation test and platelet count were normal. The presence of anti-MDA5 and anti-Ro52 antibodies was detected by Euroline immunoblotting assay. Pulmonary HRCT revealed bilateral ILD changes. Pulmonary function tests showed a mild restrictive ventilatory defect (FVC predicted: 63.8%). A diagnosis of MDA5^+^DM complicated by ILD was made. She received a combined immunosuppressive treatment of intravenous methylprednisolone (80 mg per day) and cyclosporine (75 mg twice daily), which contributed to prompt improvement of myalgia and cough.

Fifteen days after admission, she complained of unexpected right abdominal pain without traumatic event or anti-thrombotic treatment. The HB level dropped to 92 g/L, and abdominal CT showed new-onset hematoma of the right musculi obliquus internus abdominis. Fortunately, the hematoma did not expand, and vital signs of the patient were stable after hemostatic therapy and supportive treatment. She made a successful recovery and was discharged after a short period of rehabilitation. She has been continuously followed up with stable disease until now in our center.

In addition, another five cases of MDA5^+^DM complicated by SIH were identified in our MDA5^+^DM cohort, with an overall incidence of 1.1% (7/635). Detailed case description and key information are summarized in Supplementary Material and Table 1. The protocol of this study was approved by the ethics committees of Renji Hospital. Written informed consent was obtained from each patient or immediate family member.

**Table 1:** Reported cases of spontaneous intramuscular hemorrhage in patients with dermatomyositis in published studies and our cohort.

**Case**	**Age (years)**	**Sex**	**DM Duration (month)**	**Hematoma location**	**CK (U/L)**	**Coagulation function**	**PLT (**×10^9^)****	**ANA**	**Myositis specific and associated antibodies**	**High-dose glucocorticoid therapy**	**Prophylactic anti-thrombotic drug**	**Outcome**	**Reference**
1	50	F	192	Retroperitoneum	161	Normal	240	NA	NA	None	None	Survival	(8)
2	11	F	18	Right sternocleidomastoid, left rectus sheath	NA	NA	NA	1:320 (speckled)	Negative	Yes	None	Survival	(8)
3	80	M	NA	Right thigh, left oblique, left rectus sheath	1850	APTT prolonged	Normal	NA	NA	Yes	Yes	Survival	(9)
4	65	F	1	Retroperitoneum	440	PT prolonged	384	1:320	Negative	Yes	None	Death	(10)
5	77	F	1	Right sternocleidomastoid, left rectus sheath	302	PT prolonged	Normal	Negative	Negative	Yes	Yes	Death	(11)
6	64	F	4	Right psoas and iliacus, left rectus sheath	432	Normal	Normal	Positive	NA	Yes	Yes	Death	(12)
7	65	F	1	Bilateral iliopsoas	6443	APTT prolonged	80	Negative	Negative	Yes	Yes	Survival	(13)
8	60	M	1	(Left deltoid) trapezius	807	APTT prolonged	NA	1:80 (speckled)	Ro52	Yes	Yes	Death	(7)
9	50	M	1	Right iliacus and psoas	NA	NA	NA	NA	Ro52	Yes	NA	Death	(14)
10	50	M	24	Retroperitoneum, right iliacus and psoas	NA	NA	NA	Negative	Ro52	Yes	NA	Death	(15)
11	63	F	12	Retroperitoneum, right pectineus, right iliopsoas	NA	NA	176	Negative	Ro52	Yes	Yes	Death	(15)
12	24	M	NA	Bilateral brachial	Normal	NA	NA	NA	MDA5	None	NA	Survival	(16)
13	60	F	NA	Left psoas	955	NA	NA	NA	NA	NA	Yes	Death	(17)
14	64	F	5	Right pectoralis major, left anterior thigh	NA	NA	NA	NA	Mi-2	Yes	NA	Survival	(18)
15	53	F	NA	Left iliopsoas	Elevated	NA	NA	Positive	NA	Yes	Yes	Death	(19)
16	35	M	1	Lower limbs	17711	NA	NA	1:640 (speckled)	NXP2, Ro52	Yes	NA	Survival	(20)
17	41	M	1	Right iliopsoas and psoas	978	Normal	119	Negative	MDA5	Yes	Yes	Death	Our case
18	66	F	2	Right musculi obliquus internus abdominis	344	Normal	186	Negative	MDA5, Ro52	Yes	None	Survival	Our case
19	39	F	8	Retroperitoneum	5	Normal	239	1:100	MDA5	Yes	Yes	Death	Our case
20	58	M	1	Right iliopsoas and left gluteus maximus	3126	Normal	93	1:320	MDA5, Ro52	Yes	None	Survival	Our case
21	43	F	2	Right iliopsoas	1462	Normal	77	1:100	MDA5, Ro52	Yes	None	Death	Our case
22	55	F	1	Right Pectoralis, left iliopsoas and psoas	1750	Normal	155	Negative	MDA5, Ro52	Yes	None	Death	Our case
23	55	F	2	Left iliopsoas and psoas	84	Normal	134	1:40	MDA5, Ro52	Yes	None	Death	Our case

*NA, not available; M, male; F, female; DM, dermatomyositis; PT, prothrombin time; APTT, activated partial thromboplastin time; ANA, antinuclear antibody; CK, creatine kinase; PLT, platelets count. High-dose glucocorticoid therapy stands for >= 1 mg prednisone per kilogram per day; prophylactic anti-thrombotic drugs include aspirin, unfractionated heparin, and low-molecular-weight heparin*.

### Literature Search Strategy and Statistics

We used the PubMed, Embase, Web of Science, Chinese National Knowledge Infrastructure (CNKI) and Wanfang databases to search for studies describing patients with spontaneous hemorrhagic myositis in the context of a previous or concurrent diagnosis of DM. The search was conducted using the following terms: “dermatomyositis” and “hematoma” or “hemorrhage” or “spontaneous hemorrhage” or “spontaneous hemorrhagic myositis.” The search was performed from the inception of each database to July 2021.

Articles that did not clearly report the outcome of patients and articles that described hemorrhagic myositis in patients without DM diagnosis were excluded. Two researchers reviewed the search results and agreed on the included articles. The flowchart for detailed case-identifying process is shown in Supplementary Figure.

Data of demographic information, disease characteristics, treatment, and outcome were collected as detailed as possible from the included studies. Baseline characteristics and treatment data of the patients were compared between the deceased and survivors by Student's *t*-test, Mann-Whitney U tests, and Fisher's exact test, as appropriate. Parameters with >25% missing data were excluded. Statistical analyses were performed using SPSS V.23 (Armonk, NY, United States). Significance was defined as *p* < 0.05.

## Results

A total of 16 eligible DM cases with spontaneous intramuscular hematoma were identified from 14 previously published studies based on the above search strategy and exclusion criteria. Clinical and laboratory features and outcomes of 23 patients (including our cases) are summarized in Table 1.

Cumulatively, 14 patients (60.9%) died among all the 23 cases. Eleven patients (78.6%) died of an SIH-related complication (e.g., hemorrhagic shock, DIC, sepsis). Two other patients died of ILD deterioration due to underlying MDA5^+^DM. The remaining one died of treatment-related severe infection. The patients were predominantly female (65.2%) with a median age of 55 years on admission. The median disease duration of DM on admission was 2 months (*n* = 19). Fourteen out of nineteen (73.7%) patients developed SIH within 6 months of DM onset. Median creatine kinase was 881 U/L (*n* = 16). MSAs results were recorded in ten patients, 80% of which had an anti-MDA5 antibody. Besides, one case with positive anti-NXP2 antibody and another case with anti-Mi2 antibody were also documented.

**Figure 2:**
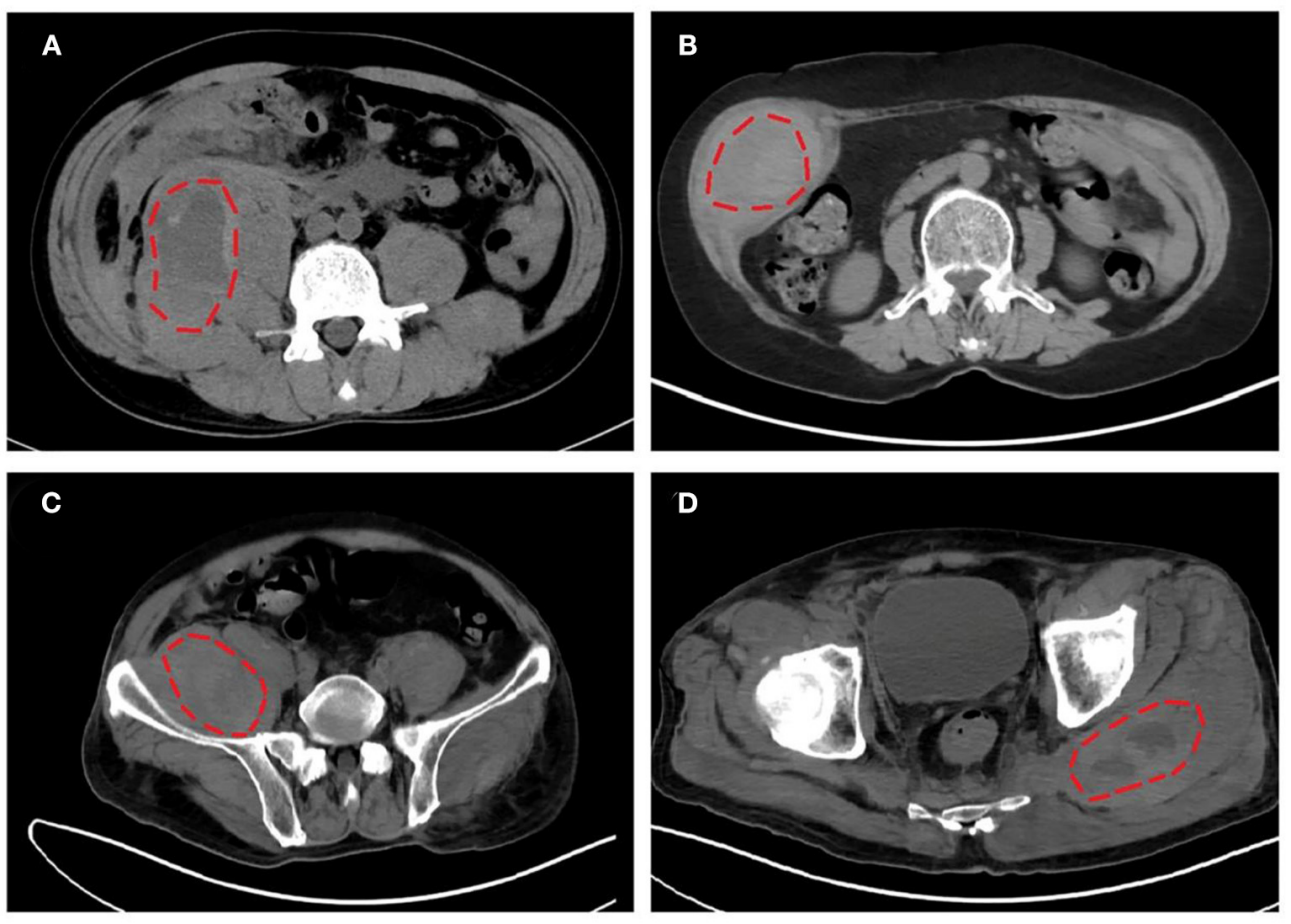
Representative computed tomography (CT) images of intramuscular hematomas in our cases. **(A)** Hematoma in right iliopsoas and psoas of listed case no. 17. **(B)** Hematoma in right musculi obliquus internus abdominis of listed case no. 18. **(C,D)** Hematomas in right iliopsoas and left gluteus maximus of listed case no. 20.

Iliopsoas (including psoas and iliac muscles, 52.2%, 12/23) was the most frequently involved bleeding location, followed by limb girdle muscles (26.1%, 6/23), retroperitoneal muscles (21.7%, 5/23), and rectus sheath muscles (21.7%, 5/23). Representative CT images of intramuscular hematomas of our cases are shown in Figure 2. The locations of hematoma could be briefly categorized into non-palpable deep muscles (e.g., iliopsoas and retroperitoneal muscles) and palpable superficial muscles (e.g., limb girdle muscles and rectus sheath muscles).

Univariable comparisons of clinical characteristics and treatment data between the deceased and survivors are summarized in Table 2. Age and sex ratio of the patients were comparable between the two groups. The median disease duration of DM was 1.5 and 2. months in the deceased and survivors, respectively, when SIH occurred (*p* =0.65). The proportion of deep muscular hematoma was significantly higher in the deceased than the survivors (85.7 vs. 33.3%, *p* =0.023). Namely, the mortality of patients with deep muscular hematoma was 80% (12/15), significantly higher than that of patients with only superficial muscular hematoma (25%, 2/8) (*p* = 0.023**)**. The incidence of anti-Ro52 antibody was 55.6% (10/18), with no significant difference between the deceased and survivors (63.6 vs. 42.9%, *p* = 0.631). Prophylactic anti-thrombotic drugs were prescribed more in the deceased (66.7%, 8/12) than in the survivors (33.3%, 2/6), although non-significantly (*p* = 0.321). The percentage of high-dose glucocorticoid use was comparable between the two groups (100 vs. 77.8%).

**Table 2:** Comparisons of clinical characteristics and treatment data between the deceased and survivors in dermatomyositis complicated by spontaneous intramuscular hemorrhage.

**Characteristics**	**Deceased (*n* = 14)**	**Survivors (*n* = 9)**	**Missing value, *n* (%)**	***p* value**
Age (years, mean ± SE)	55.4 ± 10.5	50.3 ± 22.5	0 (0)	0.545
Female	71.4% (10/14)	55.6% (5/9)	0 (0)	0.657
Disease duration (months, median, quartiles)	1.5 (1.0; 7.0)	2.0 (1.0; 18.0)	4 (17.4%)	0.650
Deep muscular hematoma (non-palpable)	85.7% (12/14)	33.3% (3/9)	0 (0)	**0.023**
Anti-Ro52 antibody	63.6% (7/11)	42.9% (3/7)	5 (21.7%)	0.631
Prophylactic anti-thrombotic drugs	66.7% (8/12)	33.3% (2/6)	5 (21.7%)	0.321
High-dose glucocorticoid	100% (13/13)	77.8% (7/9)	1 (4.3%)	0.156

*Bold values stand for statistically significant*.

## Discussion

Spontaneous intramuscular hemorrhage (SIH) is a rare (~1% in our cohort) but potentially fatal complication of DM with a strikingly high mortality (~60% in this analysis). Here, we presented the largest case series of SIH in patients with DM, specifically MDA5^+^DM. To the best of our knowledge, it is the first study to report the incidence of this complication in DM with specific MSA profile. Furthermore, based on the literature review, the potential prognostic factors of DM patients complicated with SIH were first analyzed.

It is generally known that non-palpable deep muscles (e.g., abdominal and pelvic muscles,) are not commonly involved in DM, in contrast to limb girdle muscles, even if clinically evident myositis is an atypical manifestation in MDA5^+^DM. However, in this analysis, patients with DM and deep muscular hematoma had significantly worse survival than those with superficial muscular hematoma, with iliopsoas being the most frequently involved. Based on this novel finding, we would like to draw clinicians' special attention to DM patients with deep muscles involved for the potential risk of occurrence of SIH and strikingly high related mortality. Imaging tests such as PET-CT and magnetic resonance imaging (MRI) could be useful to screen or confirm suspected myositis with or without relevant symptoms.

The majority of patients developed intramuscular hematoma in the acute phase (i.e., DM duration <6 months) in our study. These data supported the speculation of Hanawa et al. that active inflammation of the involved muscles and corresponding blood-supplying vessels might lead to SIH in DM (7).

It is widely accepted that myositis is absent or mild in MDA5^+^DM, although approximately 13% of patients could have clinically evident muscle involvement in our multicenter MDA5^+^DM-ILD cohort (1). Moreover, elevated creatine kinase values were observed in the majority of reported DM cases with intramuscular hematoma including our cases, as shown in Table 1. The phenomenon is rational that classical DM is at higher risk for this complication rather than clinically amyopathic dermatomyositis (CADM).

The recently popularized MSA detection has greatly facilitated the diagnosis, categorization, and prognostication of dermatomyositis. Unfortunately, most previously reported cases did not have MSA results except for 3 cases (1 Mi-2, 1 NXP2, and 1 MDA5), which impeded us to examine the potential correlation between MSA profile and pathogenesis or prognosis of this complication. In addition, the anti-Ro52 antibody, a myositis-associated antibody, was also found to be quite prevalent in DM, especially in MDA5^+^DM. It has also been related to higher mortality because of rapidly progressive interstitial lung disease (21). Actually, the anti-Ro52 antibody was also quite common in our analysis, with >50% incidence, although no significant difference was found between the deceased and survivors. Because of limited sample size, the current data could not firmly establish or exclude the role of the anti-Ro52 antibody in the pathogenesis or prognosis prediction of DM-related SIH. More case reports with specific MSAs and MAAs are encouraged for further investigation of this issue.

According to previous reports, the use of anticoagulation treatment has also been speculated as a probable provoking factor of hemorrhagic myositis in patients with DM (9, 11, 18). Of note, more than half of patients were receiving anti-thrombotic therapy when hemorrhagic events occurred, probably meant to prevent deep venous thrombosis. Besides, a trend toward higher percentage of prophylactic anti-thrombotic use was observed in the deceased. The statistical power might be limited by the small sample size and issue of missing value. Our data that showed the potential risk of prophylactic anti-thrombotic therapy in patients with DM should not be ignored, especially in those with non-palpable deep muscles involvement. The risks and benefits of prophylactic anti-thrombotic treatment need to be carefully balanced in routine clinical practice.

There are limitations in our study. First, because of the retrospective design of case-based review, there is an inherent missing data issue. Parameters with >20% missing values could hardly be considered for statistical analysis. Hence, we might have missed some potential prognostic effects, e.g., MSAs and creatine kinase. Second, owing to the rarity of this complication, sample size was inevitably small, which hindered us to perform multivariable analysis. Larger-scale studies are expected to validate our findings in the future.

In summary, we presented seven cases of MDA5^+^DM complicated by SIH. Based on literature review, this rare but potentially fatal complication is more likely to occur in the acute phase of DM. In addition, intramuscular hematoma in non-palpable deep muscles such as iliopsoas was probably related to gravely poor survival. Clinicians should be cautious of this tricky complication and carefully balance the risks and benefits of prophylactic anti-thrombotic treatment in daily practice. Further research is required to elucidate the pathogenetic mechanism and risk factors of spontaneous intramuscular hemorrhage in DM.

## Data Availability Statement

The original contributions presented in the study are included in the article/Supplementary Material, further inquiries can be directed to the corresponding authors.

## Ethics Statement

The patients/participants or immediate family members provided their written informed consent to participate in this study.

## Author Contributions

ZX, XL, SY, HD, and WW contributed to the study design. ZX, XL, WX, YY, XW, HD, and WW collected the clinical data. ZX and WW performed the literature review. ZX, SY, and WW contributed to the statistics analysis and data interpretation. ZX, XL, SY, and WW wrote the manuscript. All authors have revised and agreed to the final version of the manuscript.

## Funding

This work was supported by the National Youth Natural Science Foundation (grant number 81601402).

## Conflict of Interest

The authors declare that the research was conducted in the absence of any commercial or financial relationships that could be construed as a potential conflict of interest.

## Publisher's Note

All claims expressed in this article are solely those of the authors and do not necessarily represent those of their affiliated organizations, or those of the publisher, the editors and the reviewers. Any product that may be evaluated in this article, or claim that may be made by its manufacturer, is not guaranteed or endorsed by the publisher.
