# Supplementary material for: Spontaneous Intramuscular Hemorrhage in Anti-MDA5 Positive Dermatomyositis: A Case Series and Literature Review
Source: Front Med (Lausanne). 2022 Jan 24;8:802753. doi: 10.3389/fmed.2021.802753 (PMC8818869; doi:10.3389/fmed.2021.802753)
Supplement: Supplementary file 1 [file Data_Sheet_1.PDF]

Cases presentation

Listed case no.19

A 39-year-old woman presented with rashes (Gottron's papules), arthralgia, fever, dry cough and dyspnea. Laboratory test showed positive ANA and anti-MDA5 antibody, while creatine kinase (CK), coagulation tests and platelet count were normal. Pulmonary HRCT scan revealed diffuse interstitial opacities in bilateral lungs. The diagnosis of $MDA5^{+}DM$ complicated with ILD was made and the patient immediately received high-dose intravenous methylprednisolone (120mg per day) and oral tofacitinib (5mg twice daily). Low molecular weight heparin (one dose per day) was given for prophylaxis of deep vein thrombosis (DVT). One month later, a drop of hemoglobin from 111 to 70g/L was detected and subsequent abdominal CT scan revealed a new-onset hematoma in retroperitoneal muscles. After hemostatic therapy and supportive treatment, the muscular hematoma did not expand. Unfortunately, she eventually died of severe respiratory failure due to ILD deterioration.

Listed case no. 20

A 58-year-old man presented with typical DM-like rash, fever, muscular soreness and cough. The CK value was markedly elevated (3126U/L) as well as liver enzymes. Serum ferritin level was extremely high (>15000ng/ml). The coagulation test was normal while the platelet count ($93*10^{7}$/L) was slightly decreased. The presence of anti-MDA5 and anti-Ro52 antibodies was detected. Pulmonary HRCT showed diffuse bilateral interstitial diseases. A diagnosis of $MDA5^{+}DM$ was then made. The treatment of intravenous

methylprednisolone (80mg per day) and oral cyclosporine (75mg twice daily) was initiated. On the 17th day after admission, he complained of a sudden bilateral hip pain. CT scan revealed hematomas in right iliopsoas and left gluteus maximus. Fortunately, his vital sign was stable and the hematomas did not expand after hemostatic therapy and supportive treatment. Finally, he made a successful recovery and was discharged after rehabilitation.

Listed case no. 21

A 43-year-old female patient presented with fever, rashes, fatigue, sore throat and dyspnea for 2 months. The maximum CK value was 1058 U/L. Severe liver insufficiency (ALT 569U/L & AST 1006U/L) and jaundice (total bilirubin 327umol/L, direct bilirubin 185umol/L) were found on admission. Detection for hepatitis viruses were all negative. Abdominal CT scan excluded solid liver lesions and biliary obstructive disease. Pulmonary HRCT showed mild interstitial lung disease. Echocardiography revealed left ventricular systolic dysfunction (Ejection Fractions=40%). B-type natriuretic peptide (BNP) was markedly elevated (1090 pg/ml). The platelet count ($77*10^7$/L) was slightly decreased while the coagulation test was normal. Double-positivity of anti-MDA5 and anti-Ro52 antibodies was detected in MSAs and MAAs tests. The diagnosis of $MDA5^+$DM was established. The hepatic and cardiac lesions were attributed to dermatomyositis-related inflammation. High-dose intravenous glucocorticoid (maximum methylprednisolone 200mg per day) was thus administrated. On the 8th day after admission, the hemoglobin level fell from 136 to 63 g/L. A subsequent CT scan revealed a spontaneous hemorrhage in right iliopsoas. The muscular hematoma did not expand after hemostatic therapy.

Unfortunately, the patient eventually died of pneumocystis jiroveci pneumonia (PJP) and blood stream infection (Acinetobacter baumannii) two months later.

Listed case no. 22

A 55-year-old woman was admitted due to Gottron’s rashes, cough and muscular soreness. Laboratory test showed elevated CK level (1750U/L). MRI of the bilateral thighs showed active myositis. The coagulation test and platelet count were normal. Positive anti-MDA5 and anti-Ro52 antibodies were detected. Pulmonary HRCT revealed bilateral interstitial changes under the pleura and multiple patchy lesions. Thus, a diagnosis of $MDA5^{+}DM$ was made. The patient was treated with intravenous methylprednisolone (80mg per day) and oral tacrolimus (1.5mg twice daily). On the 19th day after admission, ecchymosis emerged on the right chest wall and the hemoglobin dropped from 167 to 75 g/L. Subsequent CT scan revealed hematomas in the right pectoralis, together with left iliopsoas and psoas. Emergent angiography confirmed active bleeding from right thoracic acromion artery and left lumbar artery. A successful coil embolization was performed and the patient recovered from the hemorrhagic event after blood transfusion and fluid resuscitation. Unfortunately, she eventually died of ILD exacerbation due to underlying dermatomyositis one month later.

Listed case no. 23

A 55-year-old woman was admitted due to heliotrope rash, cough and dyspnea. Laboratory test showed elevated ferritin level, normal coagulation test and PLT count,

positive anti-MDA5 and anti-Ro52 antibodies. Electroneuromyography demonstrated a mild myopathic abnormality with normal CK. Pulmonary HRCT revealed diffuse interstitial changes of both lungs. A diagnosis of $MDA5^{+}DM$ was made and she was treated with intravenous methylprednisolone 40mg per day. On the 9th day after admission, the patient developed a sudden hypovolemic shock. CT scan revealed a hemorrhage in the left iliopsoas and psoas. Emergent angiography revealed active bleeding from a branch of the iliolumbar artery. Angiographic embolization didn't successfully stop bleeding and she died two days later.

Supplementary Figure The flow chart of literature review for identifying the eligible cases

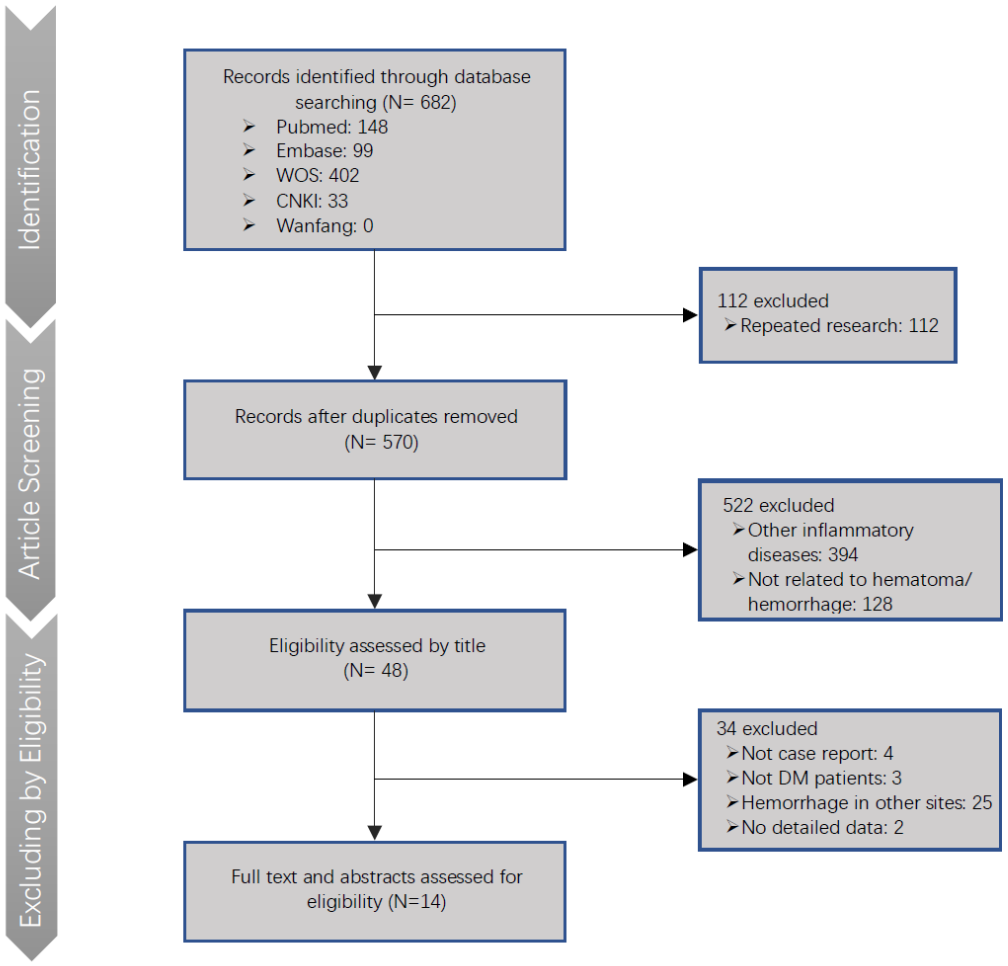

WOS, Web of Science; CNKI, Chinese National Knowledge Infrastructure
